# Exosomes derived from mir-214-3p overexpressing mesenchymal stem cells promote myocardial repair

**DOI:** 10.1186/s40824-023-00410-w

**Published:** 2023-08-10

**Authors:** Wenwu Zhu, Qingjie Wang, Jian Zhang, Ling Sun, Xiu Hong, Wei Du, Rui Duan, Jianguang Jiang, Yuan Ji, Haoran Wang, Bing Han

**Affiliations:** 1 Division of Cardiology, Xuzhou Central Hospital, Xuzhou Clinical School of Nanjing Medical University, Xuzhou Institute of Cardiovascular Disease, Xuzhou, Jiangsu China; 2 Department of Cardiology, the Affiliated Changzhou No. 2 People’s Hospital of Nanjing Medical University, Changzhou, Jiangsu China; 3 Department of Echocardiography, Zhongshan Hospital, Shanghai Institute of Cardiovascular Diseases, Shanghai Institute of Medical Imaging, Fudan University, Shanghai, 200000 China; 4 Department of Pharmacology, School of Pharmacy, Nanjing University of Chinese Medicine, Nanjing, 210023 China

**Keywords:** Exosomes, Mesenchymal stem cells, miR-214-3p, PTEN, Myocardial infarction

## Abstract

**Aims:**

Exosomes are known as nanovesicles that are naturally secreted, playing an essential role in stem-mediated cardioprotection. This study mainly focused on investigating if exosomes derived from miR-214 overexpressing mesenchymal stem cells (MSCs) show more valid cardioprotective ability in a rat model of acute myocardial infarction (AMI) and its potential mechanisms.

**Methods:**

Exosomes were isolated from control MSCs (Ctrl-Exo) and miR-214 overexpressing MSCs (miR-214^OE^-Exo) and then they were delivered to cardiomyocytes and endothelial cells in vitro under hypoxia and serum deprivation (H/SD) condition or in vivo in an acutely infarcted Sprague-Dawley rat heart. Regulated genes and signal pathways by miR-214^OE^-Exo treatment were explored using western blot analysis and luciferase assay.

**Results in vitro:**

, miR-214^OE^-Exo enhanced migration, tube-like formation in endothelial cells. In addition, miR-214^OE^-Exo ameliorated the survival of cardiomyocytes under H/SD. In the rat AMI model, compared to Ctrl-Exo, miR-214^OE^-Exo reduced myocardial apoptosis, and therefore reduced infarct size and improved cardiac function. Besides, miR-214^OE^-Exo accelerated angiogenesis in peri-infarct region. Mechanistically, we identified that exosomal miR-214-3p promoted cardiac repair via targeting PTEN and activating p-AKT signal pathway.

**Conclusion:**

Exosomes derived from miR-214 overexpressing MSCs have greatly strengthened the therapeutic efficacy for treatment of AMI by promoting cardiomyocyte survival and endothelial cell function.

**Graphical abstract:**

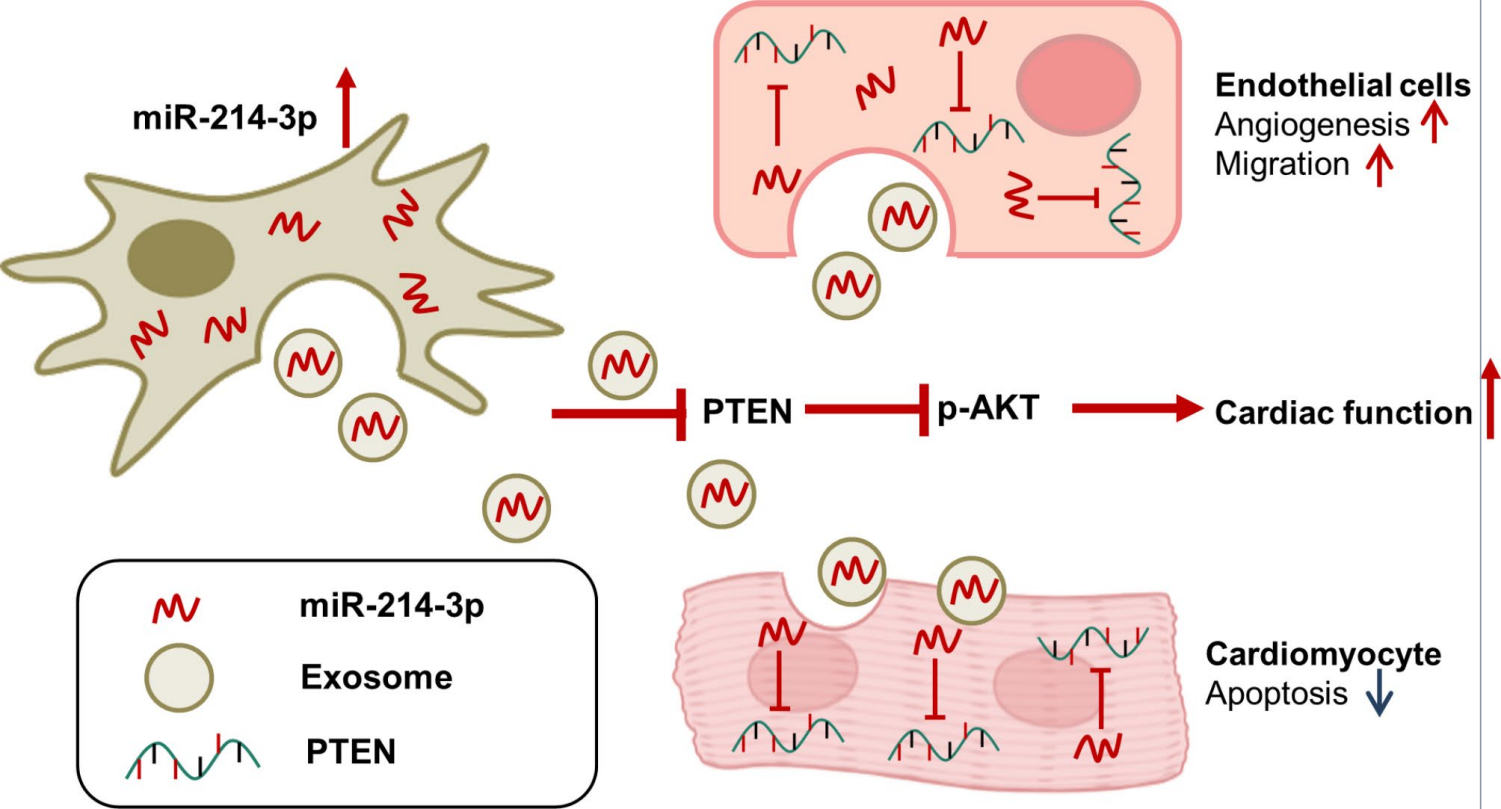

**Supplementary Information:**

The online version contains supplementary material available at 10.1186/s40824-023-00410-w.

## Introduction

Acute myocardial infarction (AMI) irreversibly causes cardiomyocytes death and myocardial remodeling, leading to ischemic heart disease [1, 2]. At present, surgical intervention and thrombolysis are important treatment methods, which may induce reperfusion injuries during and after surgical procedures, such as myocardial cell death and microvascular injury [3, 4]. It is necessary to develop effective complementary therapies to reduce the loss of cardiomyocytes.

In recent years, microRNAs (miRNAs) have been applied to treatment cardiovascular diseases [5–8]. MiRNAs controls gene-expressing by matching with 3 ‘untranslated region (UTR) of targeted mRNA, leading to mRNA degradation or translation inhibition [9]. More and more studies have shown that miRNAs take part in the regulation of myocardial angiogenesis, fibrosis and apoptosis after myocardial infarction, and become potential drug candidates for the treatment of myocardial infarction [10–12]. However, the poor stability of miRNAs in vivo and poor cellular uptake limit their clinical application [13]. Several methods have been developed for miRNAs delivery, including liposomes, viral vectors, hydrodynamic injection and nanocarriers, but these methods are related to carrier toxicity, low delivery efficiency and adverse immune reactions. Therefore, there is a great need for reliable and efficient delivery methods.

Exosomes are a group of cell-secreted heterostructures, ranging in diameter from 30 to 150 nm [14]. The lipid membrane structure of exosomes is similar to that of liposomes. Thus, exosome can protect nucleic acids from degradation, and facilitate transport of miRNAs, mRNAs, and proteins to receptor cells with no virulence and adverse immunogenicity [15–18]. Genetic engineering of miRNA in exosomes can further enhance the protective effects on different diseases [19, 20]. Therefore, exosomes may be a better alternative for the miRNA delivery in vivo. It is reported that exosomes from cardiac progenitor cells (CPCs), mesenchymal stem cells (MSCs), induced pluripotent stem cells and embryonic stem cells exert protective effects during cardiac repair as a cell-free therapy [21–23]. Compared with other stem cells, Mesenchymal stem cells (MSCs) have the advantages of easy isolation, easy amplification, low pluripotency, and immunogenicity. The exosomes secreted by mesenchymal stem cells have a higher degree of safety, tolerance and non-toxicity [24].

MiRNA-214 mediates anti-apoptosis and therapeutic angiogenesis. Lv et al. reported that miRNA-214 inhibited apoptosis of infarcted cardiomyocytes via PTEN/Akt by targeting PTEN, and promoted endothelial cell migration and angiogenesis by targeting ATM [25, 26]. In this study, we first isolated miRNA-214-rich exosomes (miR-214^OE^-Exo) from genetically modified mesenchymal stem cells, and then delivered these miR-214-exosomes to the heart tissue via intra myocardial injection in order to promote cardiac function recovery. The exosome-mediated specific miRNAs delivery might have the possible effect on the treatment of AMI.

## Methods

### Ethical statement

Animal experiments were conducted according to the Guidelines for the Care and Use of Laboratory Animals and were approved by Ethics Committee of Nanjing Medical University. Surgical operations are performed under anesthesia by sodium pentobarbital (50 mg/kg) injecting intraperitoneally.

### Cell culture and identification

Human umbilical mesenchymal stem cells (HuMSCs) were gained from Clinical Center of Reproductive Medicine in Nanjing. HuMSCs were cultured with Eagle’s Minimum Essential Medium (EMEM, Gibco, USA) containing 10% fetal bovine serum(FBS, Gibco, USA), 5ng/ml epidermal growth factor and 5ng/ml alkaline fibroblast growth factor. Human umbilical vein endothelial cells were cultured in Dulbecco modified eagle medium (DMEM, Gibco, USA) containing fetal bovine serum (10%), 100 µg/ml streptomycin, 100 U/ml penicillin, and 110 mg/ml sodium acetone. All those cells were cultured in an incubator which contains 5% CO_2_ at 37℃.

HuMSCs used for experiments were between passages 3 and 6. Oil red staining or alcian blue were used to examine differentiation capacity of HuMSCs for adipogenesis and chondrogenesis.

### Neonatal rat cardiomyocytes (NRCMs) isolation and culture

Myocardial cells were extracted from those 1-3-days old suckling rats. Ventricles were cleaned and shredded using 1× ADS (NaCl 68 g/L, HEPES 47.6 g/L, Na2HPO4 1.38 g/L, Glucose 6 g/L, KCl 4 g/L and MgSO4 1 g/L) buffer solution with pH 7.35-7.45. After that, tissues were digested by 0.6 mg/ml pancreatin and 0.4 mg/ml collagenase II. Collect the supernatant and add one-fifth horse serum of the volume of supernatant after each digestion. Finally, the cells were purified and cultured in DMEM consisting of 10% horse serum, 5% fetal bovine serum, 100 µg/ml streptomycin and 100 U/ml penicillin, and placed in an incubator with a CO_2_ content of 5% and a temperature of 37 °C. For hypoxia and serum deprivation conditions, cells were maintained without serum in a hypoxia chamber at 0.5%CO2, and 37℃ for 12 h.

### Extraction and identification of exosomes

Isolation of exosome was proceed as described previously [10]. Briefly, huMSCs with 80% confluence were washed with PBS and then cocultured in exosome free medium for 48 h. The supernatant were centrifuged at 1500 g for 30 min and incubated with Exosome Isolation Reagent (C10130-2, RiboBio, China) for 12 h at a temperature of 4 °C, and centrifuged at 2000 g for half an hour. The precipitates were suspended with PBS and stored at 80 ℃. Bicinchoninic acid assay (BCA, Thermo Fisher Scientific, Waltham, MA) was used to detect exosomes protein concentration by measuring absorbance at 562 nm. Transmission electron microscopy (TEM; Hitachi H-7650; Japan) was used to observe morphology of exosomes and western blot was carried out to detect surface markers CD81, CD63 and TSG101. Nanoparticle Tracking Analysis (NTA) was used to analyze the size and concentration of exosomes.

### Lentiviral constructions and infection

The lentivirus was gained from Shanghai Genechem Co., LTD. There are two types of lentiviral recombinant vectors. One is Ubi-MCS-SV40-EGFP-IRES-puromycin, used as hsa-miR-214-3p^OE^ (overexpression of hsa-miR-214-3p) lentivirus. Another is H1-MCS-CMV-EGFP, used as hsa-miR-214-3p^NC^ (Control) lentivirus. HuMSCs were incubated in 24-well plates, grow to 50% confluence, and infected with hsa-miR-214-3p^OE^ virus or hsa-miR-214-3p^NC^ virus, respectively. The fluorescence signals were observed and the expression of hsa-miR-214-3p was detected by qRT-PCR.

### Exosome uptake assay

Exosomes were labelled by 1 µmol Dil (ThermoFisher, USA) and were cultured with cells for 6 or 24 h in vitro. Nuclei were stained with 4′,6-diamidino-2-phenylindole (DAPI, Beyotime, Shanghai, China). Zeiss laser-scanning confocal microscope (LSM5 Live, Carl Zeiss, Germany) were used to evaluate the absorbance of exosomes.

### Migration assay

HUVECs were planted on 6-well plate with 2 × 10^5^ cells/well. Cells were treated with 1 mL test medium for 24 h. After that, cells were rinsed and scratched by P200 pipette tip. Cell migration was evaluated 24 h later. All samples were observed with three replicates.

### Tube formation of HUVECs assay

Capillary tube formation assay was used to observe angiogenesis of HUVECs. HUVECs were incubated in 96-well plate coated with growth factor reduced Matrigel (356,230; BD Biosciences, SanJose, CA, USA). Six hours later, formation of capillary-like tubes was observed and the tube length was analyzed by Image J software. All experiments were carried out with three replicates.

### Immunofluorescence and TdT-mediated dUTP nick‐end labeling staining

Cardiomyocytes were fixed with 4% paraformaldehyde for 15 min, and then permeabilized with 0.5% Triton X-100 for 15 min. After that, cells were incubated with 10% goat serum for 2 h, α‐actinin primary antibody (1:150; A7811; Sigma, Santa Clara, CA) overnight at 4℃ and second antibody for 1 h at room temperature. TdT‐mediated dUTP nick‐end labeling (TUNEL) staining was used to examine apoptotic cells following manufacturer’s instructions (Roche, Mannheim, Germany).

### MI model establishing and exosomes injection

Sprague Dawley rats (Male, 6–8 weeks) were provided by animal center of Nanjing Medical University (Nanjing, China). Rats were injected 50 mg/kg sodium pentobarbital and mechanical ventilated via orotracheal intubation. The left anterior descending coronary artery was ligated 1.5 mm at the lower edge of the left auricle by thoracotomy. Exosomes (50ug) were injected into 4 portions at the border of the infarction area. All surgeries and analyses were blind interventions.

### Cardiac function assessment

The cardiac function was measured by transthoracic echocardiography 28 days after exosome injection with VEVO 2000 high-resolution micro-imaging system. A 30 MHz transducer was used to measure the left ventricular short-axis in M-mode. The left ventricular ejection fraction (LVEF) and left ventricular shortening rate (LVFS) were calculated by Vevo2000 workstation software. LVEF is calculated from the measured values of end-diastolic volume (EDV) and end-systolic volume (ESV), and its formula is as follows: LVEF=(EDV ESV)/EDV × 100%. LVFS can be directly obtained from two-dimensional ultrasound guided M-mode images or two-dimensional images.

### Masson trichrome staining and hematoxylin-eosin staining

Heart tissues were collected, fixed and sliced to 5 μm. Mason’s trichrome staining as described above [9]. The proportion of infarct area is the sum of infarct area of each section/sum of LV area of each section × 100%. Hematoxylin-Eosin (HE) stain was used to measure the level of inflammatory cells.

### Fluorescence in situ hybridization (FISH) of miR-214

MiR-214^OE^ exosomes (50ug) were injected at the edge of the infarction area. After 6 h, the rats were sacrificed. The miR-214 oligonucleotide probes (Sangon Biotech, China, 8 ng/µL) was incubated with Cy3 or FAM in pre-hybridization buffer at 37 °C for 1 h, and then hybridized overnight at 37 °C. With Sect. 2 × SSC, 1 × SSC, 0.5 × SSC rinsing and DAPI staining, the internalization of miR-214 in recipient cells or myocardium was observed by confocal microscope.

### Immunofluorescence

Immunofluorescence was performed as reported previously [27]. Briefly, heart tissue were collected and then fixed, embedded, and sectioned. After that, the section was stained with primary antibody anti-actin and CD31 (1:200, ab7388, Abcam, Cambridge). The nuclei was stained with DAPI.

### Western blot analysis

Western blotting was performed with a standard protocol as previously described [10]. Antibodies were used as follows: GAPDH (1:1000; 5174 S; Cell Signaling Technology), Bcl-2 (1:1000; 2870 S; Cell Signaling Technology), AKT (1:1000; 4961 S; Cell Signaling Technology), p-AKT (1:1000; 4060 S; Cell Signaling Technology), BAX (1:1000; 5023, Cell Signaling Technology), PTEN (1:1000; ab31392, Abcam).

### Quantitative real-time polymerase chain reaction analysis

Total RNAs were extracted by Trizol and treated with RNase-free DNase I (1/20 µL, Promega Corp, Madison, WI). Reverse transcription of cDNAs was performed using Reverse Transcription Kit (Takara, Dalian, China). Polymerase chain reaction analysis (PCR) analysis was performed with SYBR green PCR Master Mix (Applied Biosystems, Foster, CA, USA) on ABI‐7900 Real‐Time PCR Detection System (7900HT; Applied Biosystems). The exosomal level of miR-214-3p was normalized to that of cel-miR-39 (C39). The cellular miR-214-3p expression was normalized to U6. The cellular expression of PTEN mRNA was normalized to that of glyceraldehyde3-phosphate dehydrogenase (GAPDH). The related gene expression was normalized to that of GAPDH. The primer sequences are listed in Additional file: Table S1.

### Flow cytometry analysis

Apoptosis of HUVECs were examined by flow cytometry using Annexin V Alexa Fluor647/PI/Apoptosis detection kit (Fcmacs Biotech, Nanjing, China). Briefly, cells were washed, digested and resuspended in a binding buffer with double distilled water at a ratio of 1:3, incubated by 5 µl Annexin V and 10 µl propidium iodide (PI) for 15 min at room temperature in the dark. After that, 400 µl PBS was added and flow cytometry analysis was performed on FAC Scan. Data were analyzed by FlowJo software.

### Luciferase assay

The PTEN 3’UTR cDNA sequences containing mir-214-3p binding site was inserted into pmir-GLO-promoter vector (Promega, Madison, USA). HEK-293T cells were transfected with miR-NC or miR-214-3p mimics and seeded into 96-well plates. The sequences of miRNAs transfected were shown in Additional file: Table S2. After that, cells were co-transfected with 100 ng pmiR-GLO-PTEN-WT or pmiR-GLO-PTEN-MUT. The luciferase activity was detected by EnSpire® 2300 Multimode Plate Reader (Perkin Elmer Singapore Pte. Ltd., Singapore) 24 h after transfection. luciferase activities of firefly were normalized to Renilla luciferase activity.

### Statistical analysis

All data are expressed in the way of mean ± standard deviation. The two-tailed test was used to compare the means between the two groups, and one-way analysis of variance was used for multiple experimental groups. SPSS statistical software (version 17.0, SPSS Inc., Chicago, IL) was used for statistical analysis. P value of less than 0.05 was considered statistically significant.

## Results

### Characterization of huMSCs and exosomes derived from huMSCs

**Fig. 1:**
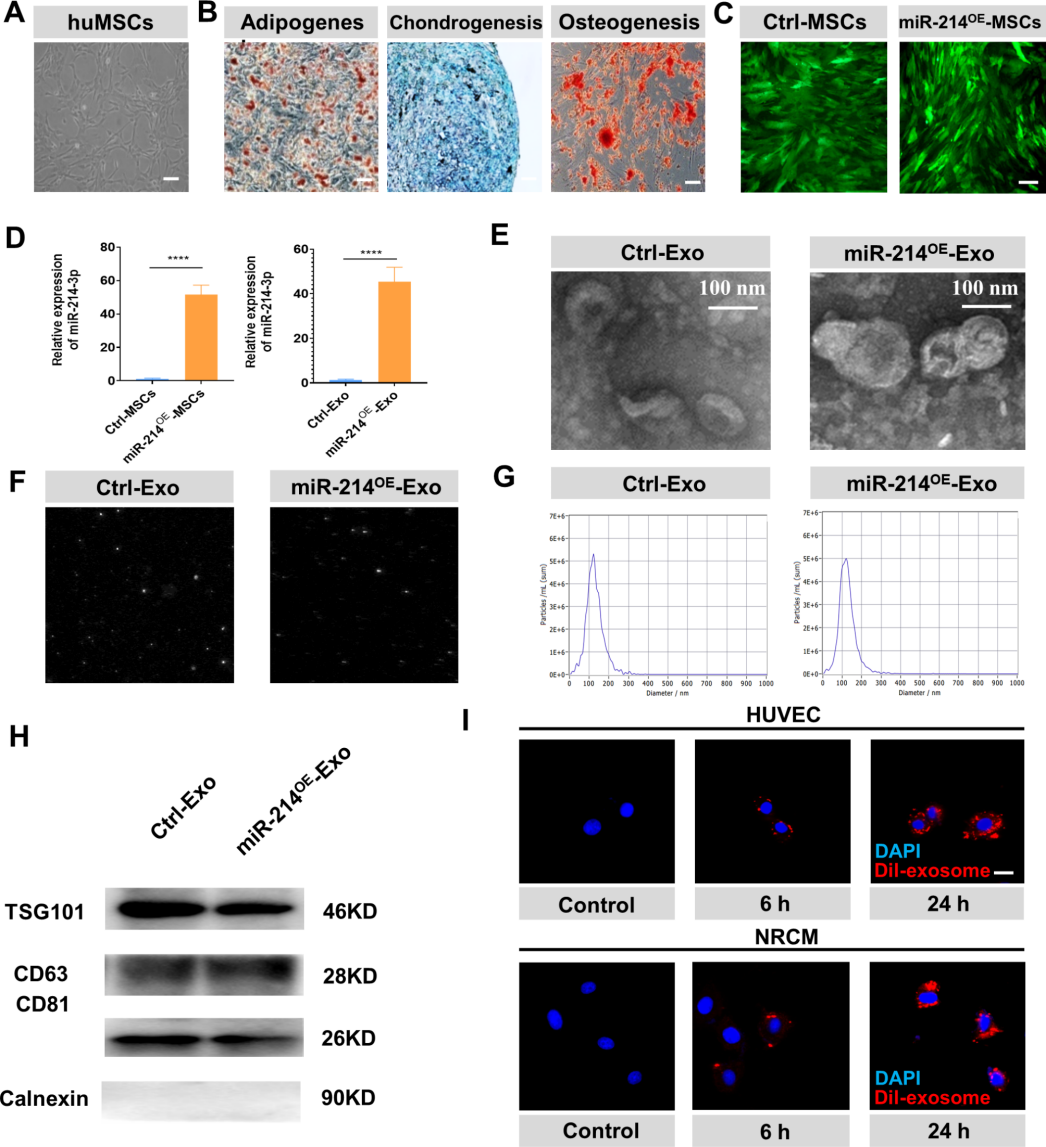
Characterization of huMSCs and exosomes which are derived from huMSCs. **(A-B)** Morphology and multiple differentiation potential of huMSCs. Scale bar = 100 μm. **(C)** Fluorescence microscopy image of lentivirus transfection in Ctrl-MSC and miR-214^OE^-MSCs groups. Scale bar = 20 μm. **(D)** The expression of miR-214-3p in cells and exosomes was verified by real-time PCR. (n = 3 biological replicates for each group). **(E)** Cup-shaped morphology of purified Ctrl-Exo and miR-214^OE^- Exo assessed by TEM. **(F)** NTA representative screen shot video; the bright white point represents a particle movement. **(G)** The particle size distribution and particle concentration were analyzed by nanoparticle tracking analysis. **(H)** The exosomal protein markers of TSG101, CD63, and CD81 in Ctrl-Exo and miR-214^OE^-Exo groups. **(I)** Confocal images of Dil labeled exosomes (red fluorescence) were endocytosed by HUVECs and NRCMs 6 and 24 h after incubation. Scale bar = 20 μm

The morphology of huMSCs was shown in Fig. 1A by a light microscope. Oil red staining, alcian blue staining and alizarin red staining confirmed multiple differentiation potential of huMSCs for adipogenesis, chondrogenesis and osteogenesis (Fig. 1B). Lentiviral modifications of miR-214-MSCs (huMSCs transfected with lentiviruses containing miR-214) and Ctrl-MSCs (huMSCs transfected with lentiviruses containing miR-214 negative control) were confirmed by examining green fluorescence using fluorescent microscopy (Fig. 1C). Compared with the Ctrl-MSC group, the cellular and exosomal miR-214-3p expression in miR-214-MSC group were increased (Fig. 1D). The RT-PCR experimental results showed that the content of miR-214 in different batches of miR-214^OE^-Exo did not differ significantly, and was much higher than the content of miR-214 in Ctrl-Exo (Additional file: Figure S1A). Transmission electron microscopy scanning showed the morphology of both Ctrl-Exo and miR-214^OE^-Exo were round or disk-shaped. (Fig. 1E). Then, we analyzed the diameters of the Ctrl-Exo and miR-214^OE^-Exo using NanoSight. Most Ctrl-Exo and miR-214^OE^-Exo had a size of about 30–150 nm (Fig. 1F-G). NTA assay also showed that there no significant difference in the number of nanoparticles between Ctrl-Exos and miR-214^OE^-Exos in the same volume (Additional file: Figure S1B-C). Exosome specific markers of TSG101, CD81 and CD63 were positive in Ctrl-Exo and miR-214^OE^-Exo, Calnexin were not detected among exosome proteins (Fig. 1H). Dil-labelled exosomes were co-cultured with HUVECs and NRCMs for 6 and 24 h under H/SD. Confocal images confirmed the uptake of labeled exosomes by NRCMs and HUVECs in a time dependent manner (Fig. 1I). Cy3-miR-214-3p fluorescence were both observed in the incubated HUVECs and NRCMs (Additional file: Figure S2).

### miR-214^OE^-Exo conferred better protective effects on HUVECs and NRCMs than ctrl-exo under H/SD in vitro

**Fig. 2:**
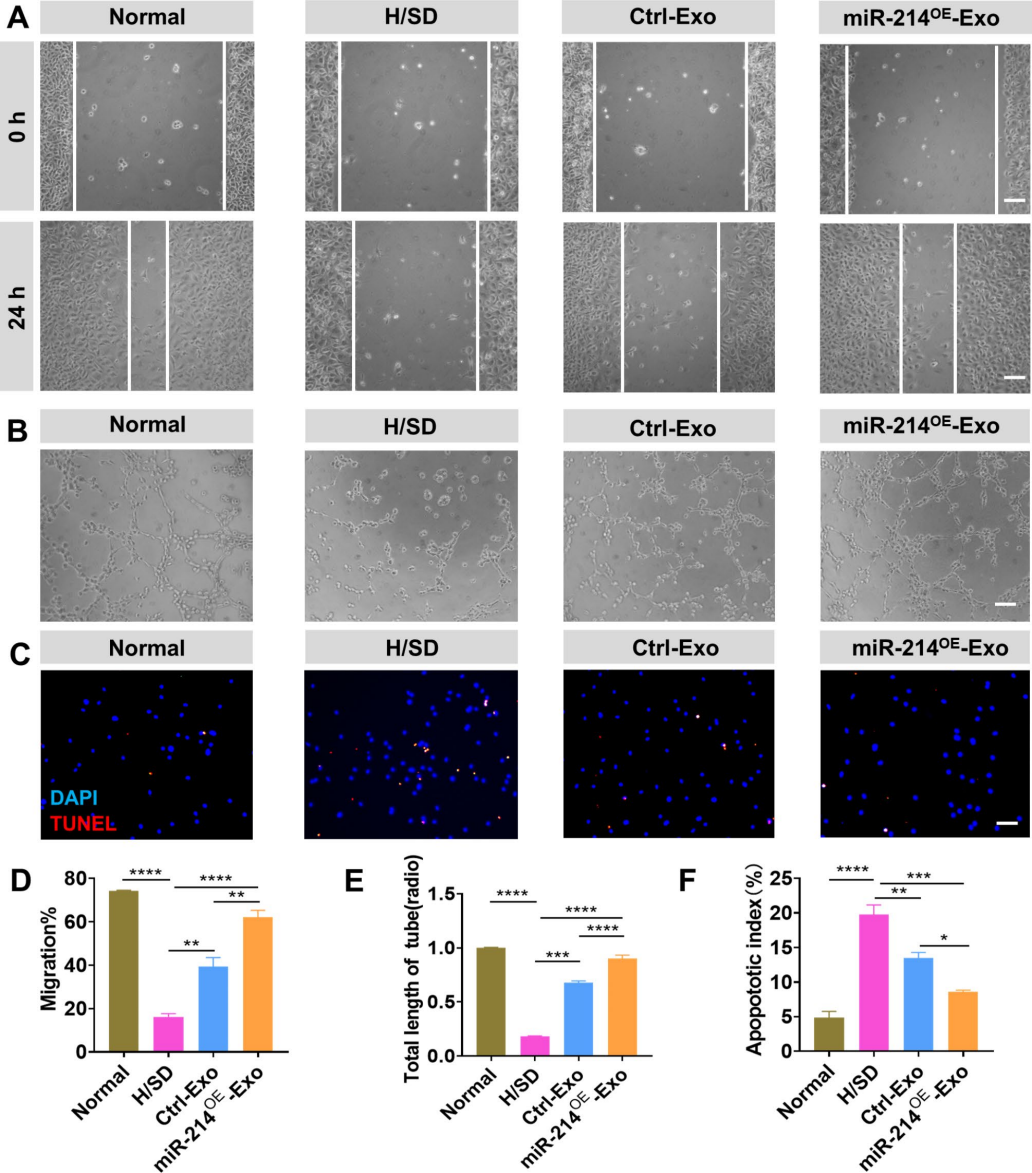
Effects of miR-214^OE^-Exo on HUVECs and NRCMs. **(A, D)** Migration of HUVECs cultured with PBS, Ctrl-Exo and miR-214^OE^-Exo was showed in Figure A & D. Scale bar = 100 μm. (n = 3 biological replicates for each group). **(B, E)** Tube formation of HUVECs incubated with PBS, Ctrl-Exo and miR-214^OE^-Exo and quantification analysis. Scale bar = 100 μm. (n = 3 biological replicates for each group). **(C, F)** Representative images of the apoptosis determined by TUNEL staining (red) in NRCMs among different groups and Quantitative analysis of the percentage of the apoptotic NRCMs among the different groups. Scale bar = 50 μm. Continuous and categorical variables were described by means ± SEM and percentages, respectively. n = 3 for each group. *P < 0.05; **P < 0.01; ***P < 0.001, ****P < 0.0001

Exosomes from stem cell exerts therapeutic benefits via promoting neovascularization. Hence, effects of miR-214^OE^-Exo on migration, tube formation, and anti-apoptosis in HUVECs and NRCMs were evaluated. Compared with PBS and Ctrl-Exo groups, migration rate of HUVECs was markedly improved in miR-214^OE^-Exo group (Fig. 2A, D). Tube formation of HUVECs also significantly increased in miR-214^OE^-Exo compared with PBS and Ctrl-Exo groups (Fig. 2B, E). TUNEL showed reduced apoptosis of NRCMs in the miR-214^OE^-Exo group compared with PBS and Ctrl -Exo groups (Fig. 2C, F). Taken together, these results showed that miR-214^OE^-Exo had cell protection effects on HUVECs and NRCMs under H/SD in vitro.

### miR-214^OE^-Exo effectively preserved cardiac function in a rat MI model

**Fig. 3:**
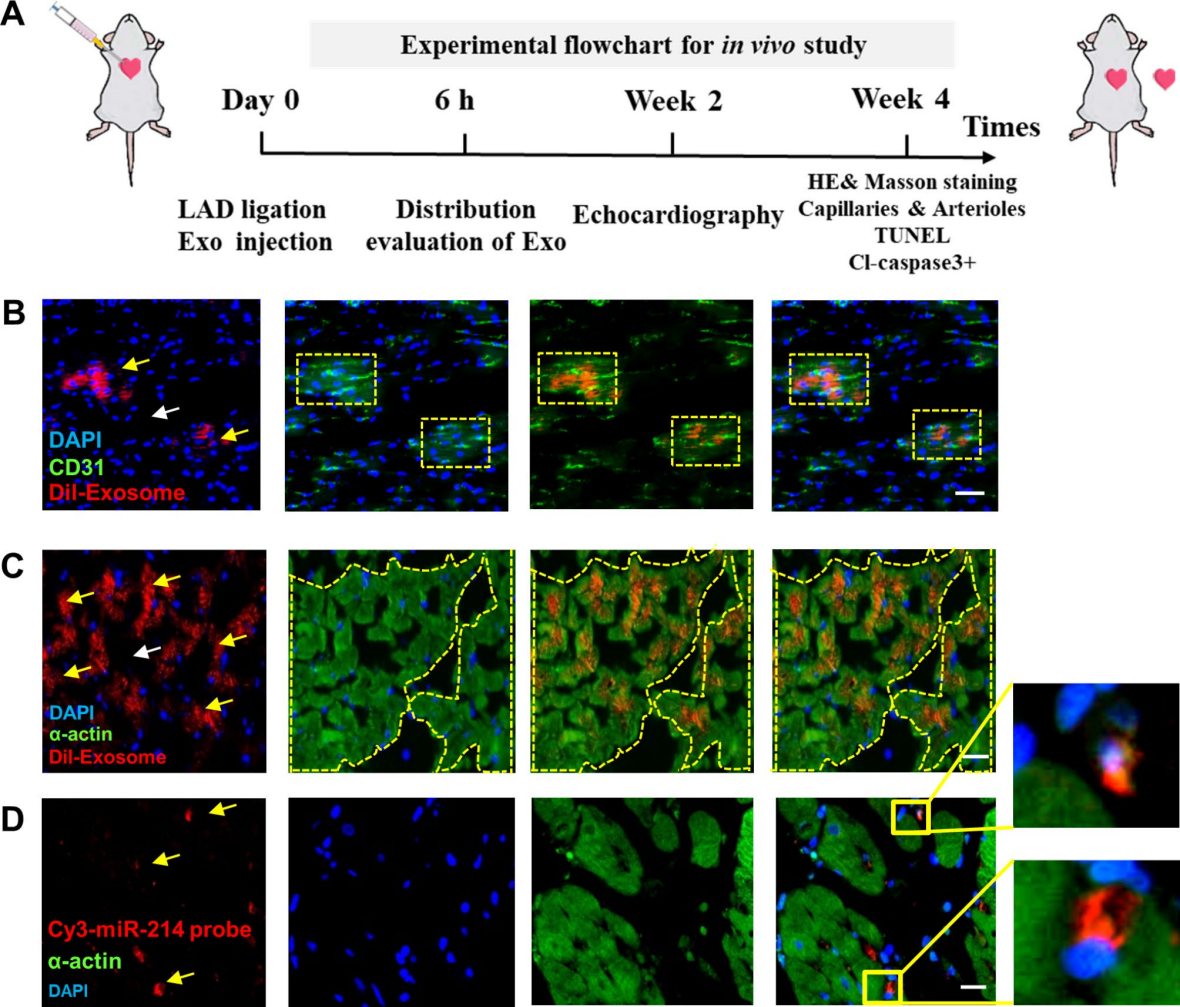
Distribution of miR-214-loaded exosomes in infarcted hearts. **(A)** Flow chart of in vivo experiment design. **(B-C)** The expression level of CD31 (B) and α-actin (C) protein was detected by immunofluorescence 6 h after intramyocardial injection of Dil labeled exosomes. The white arrow indicate the injection site. The yellow arrow indicates that exosomes were absorbed by endothelial cells and cardiomyocytes. Yellow squares indicate the region near exothelium and yellow boundaries show the region near myocardium. Scale bar = 50 μm. **(D)** FISH analysis in infarcted LV myocardium of SD Rats. Cy3-miR-214 probe (red) was used to detect miR-214. Concentrated miR-214 was indicated by yellow arrowheads. Scale bar = 20 μm

**Fig. 4:**
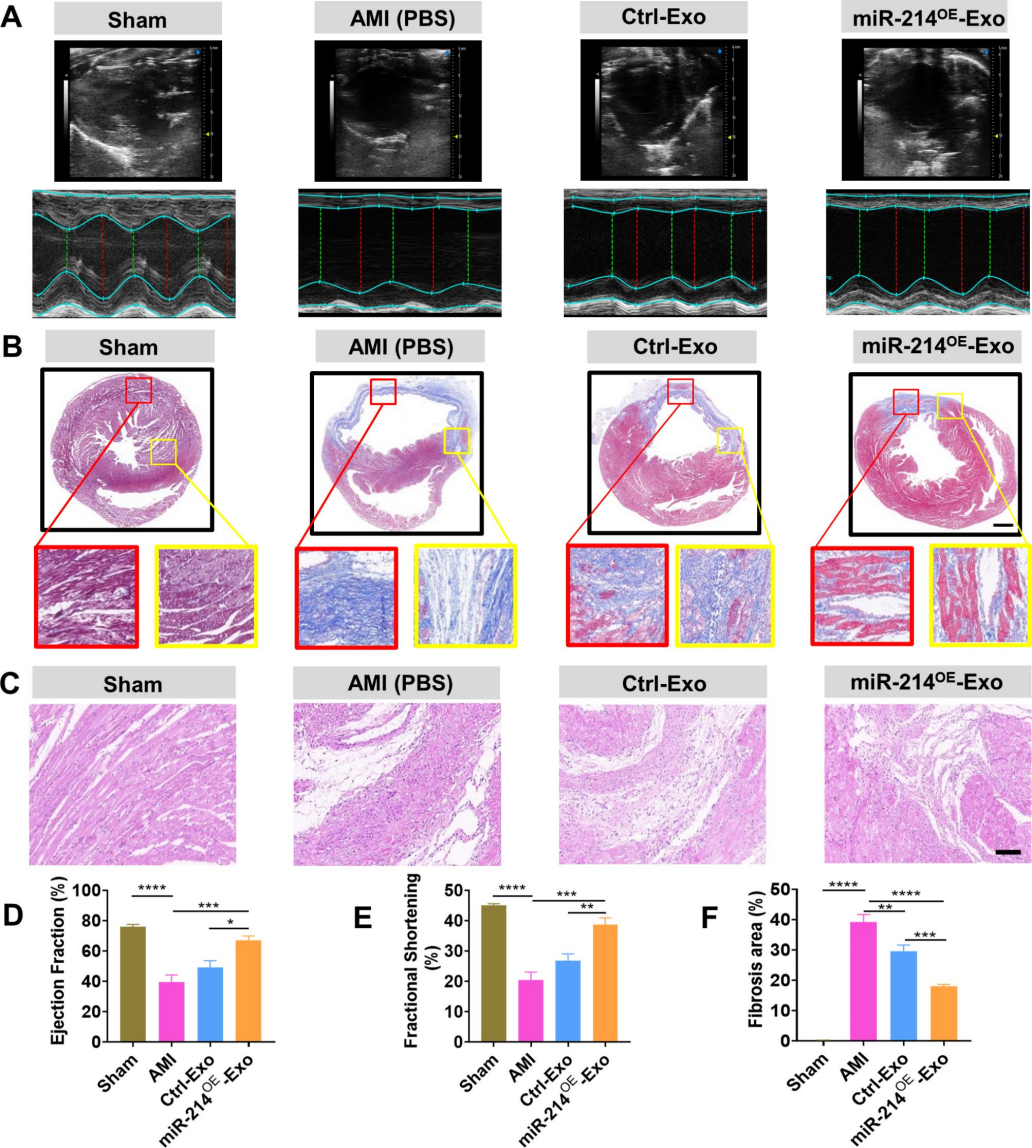
miR-214^OE^-Exo effectively preserved cardiac function in rats with MI in vivo. **(A)** Representative images of M-mode echocardiography captured 28 days after MI between different groups. **(B)** The representative Masson’s trichrome-stained myocardial cross-Sect. 4 weeks after MI with the injection of PBS, Ctrl-Exo, and miR-214^OE^-Exo. Red frame indicates the infarct zone and yellow frame indicates the border zone. Scale bar = 20 μm. **(C)** HE staining at the border zone 28 days post MI. **(D-E)** Left ventricular ejection fraction (LVEF) and left ventricular fractional shortening (LVFS) measured by echocardiography 28 days after MI. (n = 5 animals for each group). **(F)** Quantitative analysis of heart fibrosis between different groups. (n = 5 animals for each group)

MiR-214^OE^-Exo, Ctrl -Exo, or PBS (AMI control group) were injected into the border area of infarcted hearts (Fig. 3A). The distribution of Dil-labeled miR-214^OE^-Exo (Dil-Exosome) and Ctrl-Exo in the infarct heart was examined 6 h after injury. As is shown in Fig. 3, there were large Dil-labeled positive areas near exothelium (Fig. 3B) and in the myocardium (Fig. 3C), indicating that miR-214^OE^-Exo were effectively presented in myocardial cells and endothelial cells in the infarcted hearts. The quantification of incorporated exosomes in endothelial cells and cardiomyocytes was shown in Figure S3. Moreover, FISH showed that miR-214 was concentrated 6 h after injection, mainly in the peri-nuclear region of the myocardium (Fig. 3D). At 4 weeks after AMI, compared with the PBS group and the Ctrl-Exo group, LVEF and LVFS were significantly improved in the miR-214^OE^-Exo group (Fig. 4A, D, E). Masson staining showed that fibrosis area of mir-214^OE^-Exo group was significantly reduced compared with PBS and Ctrl-Exo groups (Fig. 4B, F). Also, we performed collagen immunofluorescence staining on cardiac sections and found that the level of cardiac type I collagen and cardiac type III collagen was significantly decreased after injection of miR-214^OE^-Exo (Additional file: Figure S4). Compared with the Ctrl -Exo and AMI groups, the miR-214^OE^-Exo group also had significantly less inflammatory cell infiltration (Fig. 4C). Immunofluorescence stains of iNOS + macrophages show that miR-214^OE^-Exo significantly decreased the numbers of iNOS + cells and inhibited the infiltration of inflammatory cells near the infarct area (Additional file: Figure S5). These results indicated that miR-214^OE^-Exo played a potential role in the maintenance of cardiac function in infarcted rats.

### miR-214^OE^-Exo promoted angiogenesis and cardiomyocyte survival in MI hearts

**Fig. 5:**
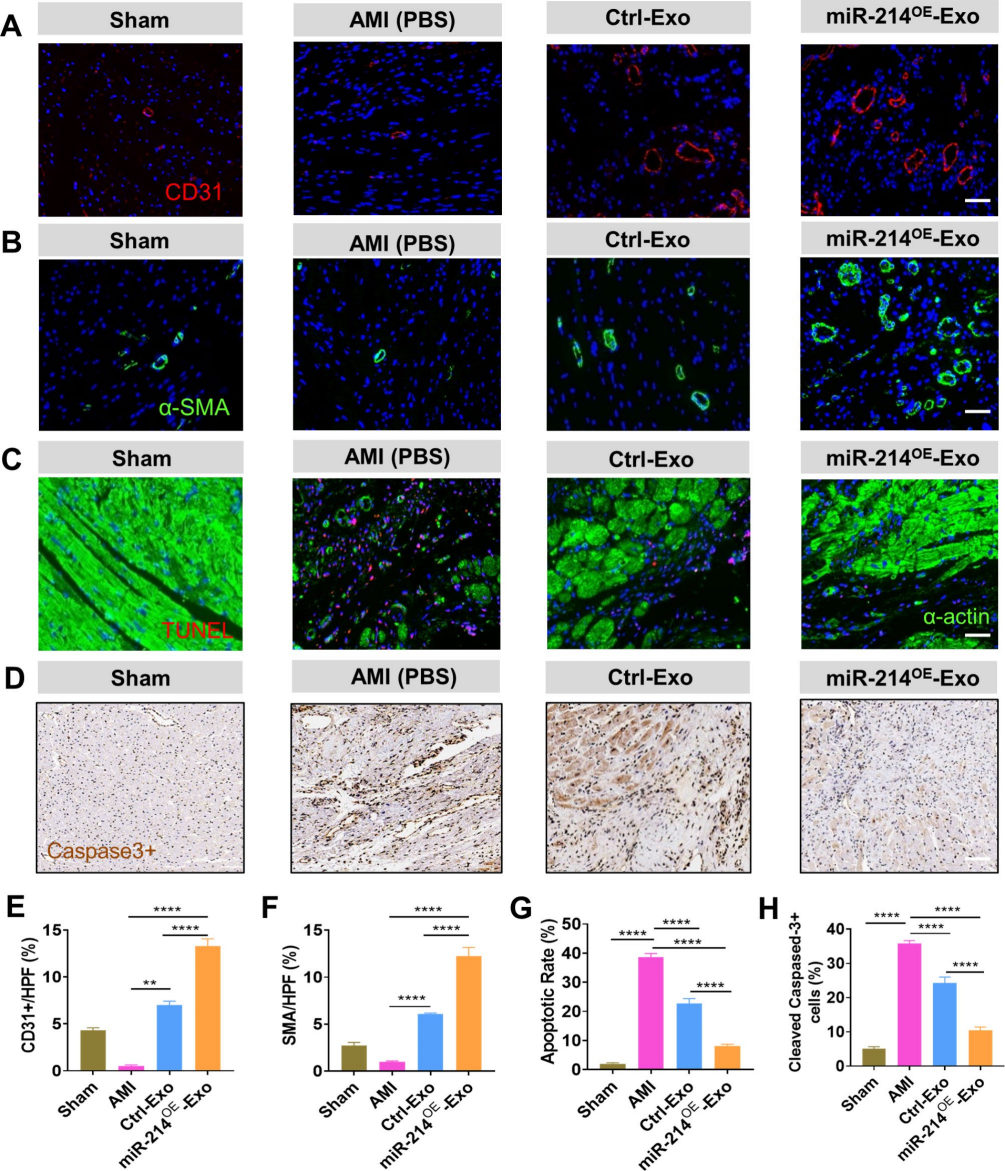
miR-214^OE^-Exo promoted angiogenesis and cardiomyocyte survival in infarcted hearts. **(A)** Neovascularization at the border zone 28 days after MI was evaluated by staining with CD31 (red) and nuclei (blue). Scale bar = 50 μm. **(B)** α-SMA positively stained arterioles at the border zone 4 weeks after MI. Scale bar = 50 μm. **(C)** Representative images showing the terminal deoxynucleotidyl transferase-mediated dUTP nick end‐labeling (TUNEL) positive cells in the heart tissue among the different groups. Scale bar = 50 μm. **(D)** Cleaved Caspase-3 staining at the border zone 28 days post MI. Scale bar = 50 μm. **(E and F)** Quantitative analysis of the CD31(E) and α-SMA(F) density in the heart tissue of rats from the different groups. (n = 5 animals for each group). **(G)** Quantitative analysis of the apoptotic rates among the different groups. (n = 5 animals for each group). **(H)** Quantitative analysis of cleaved-caspase-3^+^ cells at the border zone among the different groups. (n = 5 animals for each group)

In order to reveal the mechanism of improved cardiac function after in vitro exosome treatment, immunofluorescence staining of capillaries and arterioles were performed using CD31 and α-smooth muscle actin (α-SMA) antibodies. The results showed that the capillary density of miR-214^OE^-Exo group was significantly higher than that of Ctrl -Exo and AMI groups at 4 weeks after infarction (Fig. 5A, E). Figure 5B showed the same trends in capillary density and arteriole density (Fig. 5B, F). Figure 5 C and 5D showed the TUNEL and Cleaved Caspase-3 staining of 4-week infarcted rat hearts to revel the anti-apoptotic effect of exosomes. Compared with PBS, the number of apoptotic cells in the border zone of infarcted myocardium treated with exosomes decreased significantly. Moreover, TUNEL^+^ and Cleaved Caspase-3 ^+^ cells were minimal in the miR-214^OE^-Exo group (Fig. 5G, H). Immunofluorescent staining for WGA and Cx43 expression in in the border area post MI was conducted. We found that expression of Cx43 were significantly improved in miR-214^OE^-Exo group compared with AMI and Ctrl-Exo groups (Additional file: Figure S6). Immunofluorescence stains of α-Actinin indicated that the completeness of T-tubule was much higher in miR-214^OE^-Exo group compared with AMI and Ctrl-Exo groups (Additional file: Figure S7). These results suggest that miR-214^OE^-Exo enhances angiogenesis and cell survival, which in turn improved cardiac repair ability.

### Exosomal mir-214-3p attenuated cardiomyocyte injury via regulation of p-AKT by targeting PTEN

**Fig. 6:**
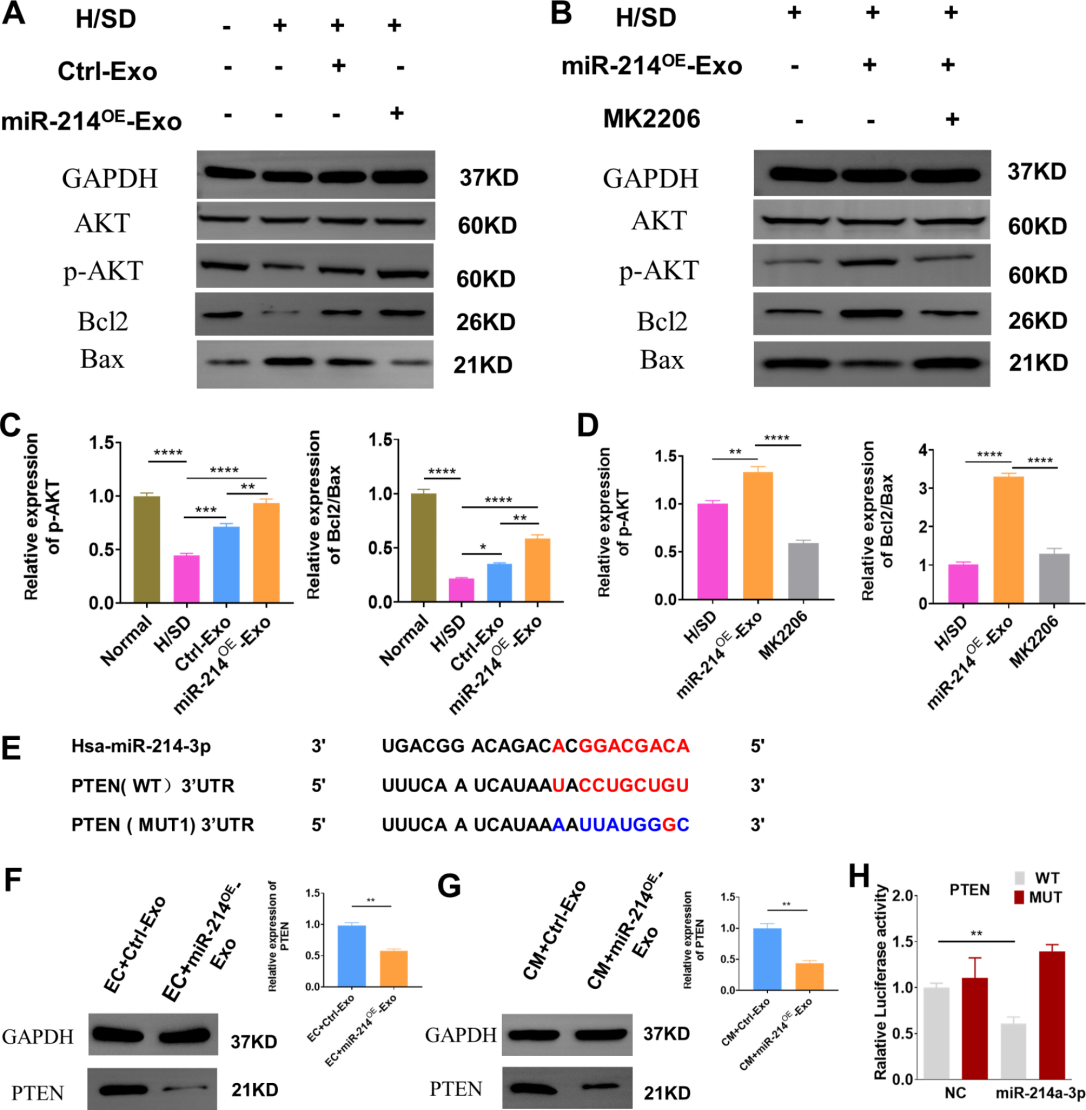
Exosomal miR-214-3p attenuated cardiomyocyte injury via regulation of p-AKT by targeting PTEN. **(A)** Western blot analysis showing the level of p-AKT、AKT、Bcl2 and Bax among the different groups. **(B)** Neonatal rat cardiomyocytes (NRCMs) were treated with miR-214^OE^-Exo or miR-214^OE^-Exo + MK2206, then exposed to H/SD. Western blot analysis showing the level of p-AKT、AKT、Bcl2 and Bax among the different groups. **(C)** Quantitative analysis of the level of p-AKT、AKT、Bcl2 and Bax among the different groups. (n = 3 biological replicates for each group). **(D)** Quantitative analysis of the level of p-AKT、AKT、Bcl2 and Bax in Fig. 6B among the different groups. (n = 3 biological replicates for each group). **(E)** The predicted miR-214 targeting sequence in the 3’-UTR of PTEN. **(F)** Western blot analysis showing the PTEN levels after administration of Ctrl-Exo or miR-214^OE^-Exo in HUVECs. (n = 3 biological replicates for each group). **(G)** Western blot analysis of PTEN after administration of Ctrl-Exo or miR-214^OE^-Exo in NRCMs. (n = 3 biological replicates for each group). **(H)** Luciferase reporter assay was performed to confirm the target gene of miR-214. (n = 3 biological replicates for each group)

Emerging evidence has revealed that the AKT signaling pathway plays an important role in regulating cell survival. Expression of p-AKT was tested to elucidate if exosomal miR-214-3p promoted cardiomyocyte survival via AKT signaling pathway. Expression of p‐AKT in NRCMs was greatly diminished under H/SD condition, whereas miR-214^OE^-Exo increased p-Akt expression under H/SD condition. Bcl2/Bax protein level was significantly higher compared with the Ctrl -Exo and AMI groups (Fig. 6A, C). AKT inhibitor (MK2206) was used to further validate the relationship between AKT and miR-214^OE^-Exo in the regulation of cardiomyocyte survival. MK2206 significantly inhibited p‐AKT induced by miR-214^OE^-Exo. In addition, MK2206 reversed miR-214^OE^-Exo induced BCL2/Bax protein levels (Fig. 6B, D). In order to further explore the mechanism of miR-214^OE^-Exo in promoting angiogenesis and anti-apoptosis, potential target genes of miR-214-3p in endothelial cells and cardiomyocytes were predicted based on the online repository of miRNA targets. We confirmed that PTEN may be a potential target for neovascularization and cardiomyocyte survival, and investigated the relationship between exosome miR-214-3p and PTEN. Figure 6E showed wild-type PTEN (Wt-PTEN) and mutant-type PTEN (Mut-PTEN) luciferase reporter gene vector. Wt-PTEN or Mut-PTEN was transfected into HEK293T cells with miR-214-3p mimics or miR-214-3p mimics NC. The transfection efficiency of the mimics and mimics NC was verified by qRT-PCR (Additional file: Figure S8A). Using Western blotting analysis, decreased expressional levels of PTEN were detected in HUVECs and NCRMs after administration of the miR-214^OE^-Exo (Fig. 6F-G). The relative luciferase activity decreased when miR-214-3p mimics were co-transfected with Wt-PTEN, but not with the Mut- PTEN (Fig. 6H). In summary, we confirmed that miR-214-3p negatively modulates PTEN by directly targeting its 3′-UTR.

### miR-214^OE^-Exo promote migration, tube formation and apoptosis inhibition by targeting PTEN

**Fig. 7:**
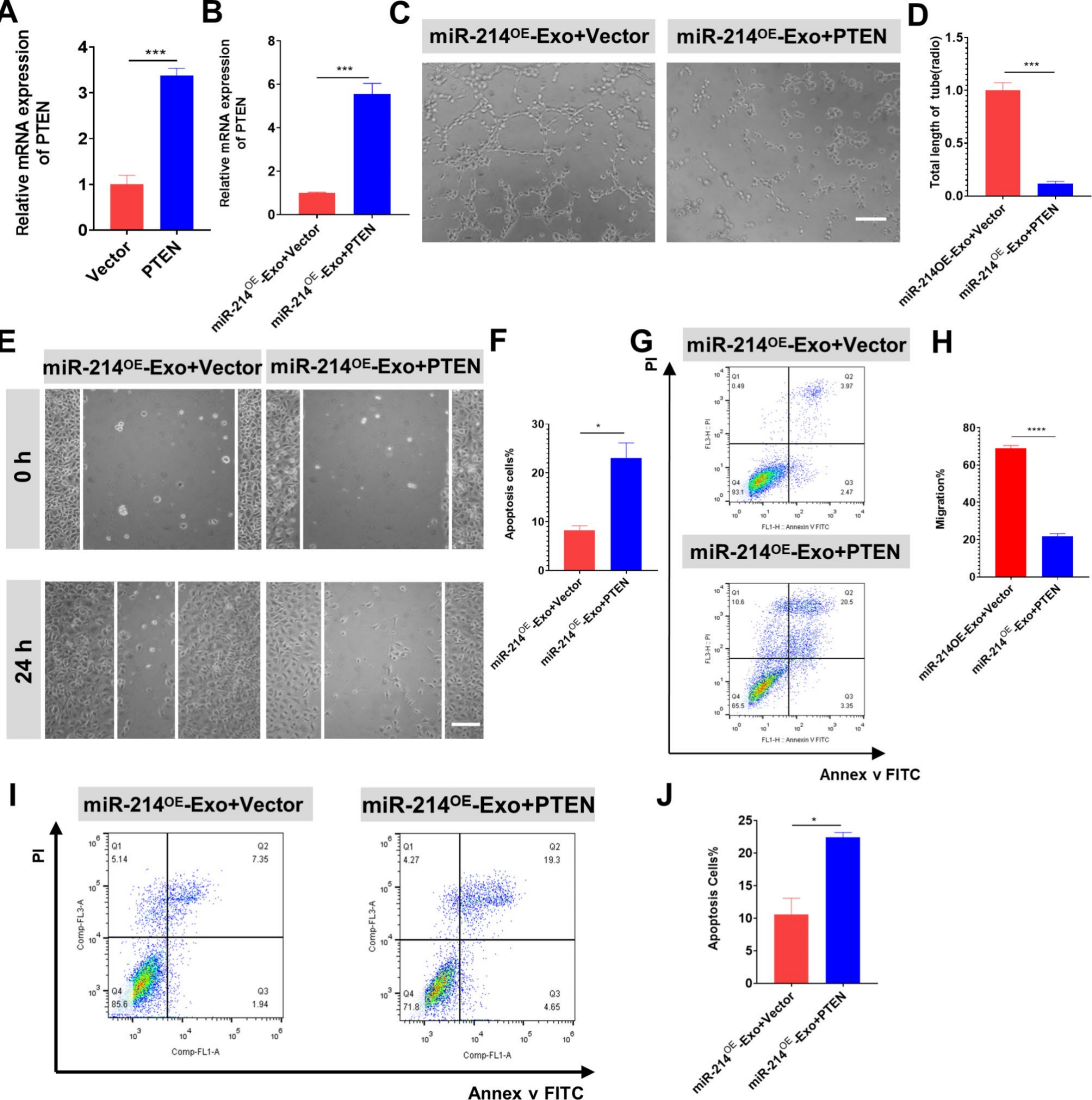
miR-214^OE^-Exo promote migration, tube formation and apoptosis inhibition by targeting PTEN. **(A)** Expression level of PTEN in HUVECs after transfection with plasmids containing Vector or PTEN. (n = 3 biological replicates for each group). **(B)** Expression level of PTEN in HUVECs after transfection with Vector or PTEN followed by administration miR-214^OE^-Exo. (n = 3 biological replicates for each group). **(C-D)** Tube formation assays were utilized to verify the functional role of PTEN on angiogenesis in HUVECs. Scale bar = 100 μm. (n = 3 biological replicates for each group). **(E-F)** Scratch assays were used to validate the functional effect of PTEN on migration of HUVECs. Scale bar = 100 μm. (n = 3 biological replicates for each group). **(G-H)** The apoptosis rate of HUVECs transfected with plasmids containing Vector or PTEN followed by administration miR-214^OE^-Exo was detected by Annexin V/FITC/PI double-staining flow cytometry. (n = 3 biological replicates for each group). **(I-J)** The apoptosis rate of NRCMs transfected with plasmids containing Vector or PTEN followed by administration miR-214^OE^-Exo was detected by Annexin V/FITC/PI double-staining flow cytometry. (n = 3 biological replicates for each group)

In vitro rescue tests were processed to investigate the association of miR-214^OE^-Exo and PTEN. PTEN and vector were inserted into HUVECs by transfection and analyzed by qRT-PCR (Fig. 7A) and Western blotting analysis (Additional file: Figure S8B). PTEN expression was also detected in HUVECs treated with miR-214^OE^-Exo. Results demonstrated that HUVECs transfected with Vector showed a significantly lower expression of PTEN when compared to PTEN after administration of miR-214^OE^-Exo (Fig. 7B, Additional file: Fig. S8C). In tube formation experiments, we observed that overexpression of PTEN inhibited angiogenesis during co-administration with miR-214^OE^-Exo (Fig. 7C-D). In addition, scratch tests showed that overexpression of PTEN eliminated the positive effects of miR-214^OE^-Exo on HUVECs migration (Fig. 7E-F). Annexin V-FITC/PI flow cytometry demonstrated that the expression of PTEN could effectively reverse the protective effect of anti-apoptosis induced by administration of miR-214^OE^-Exo (Fig. 7G-H). We also found that incubation of miR-214^OE^-Exo and PTEN could significantly lessen the effects of miR-214^OE^-Exo on anti-apoptosis in NRCMs compared with miR-214^OE^-Exo and Vector (Fig. 7I-J). Therefore, we concluded that exosomal miR-214-3p enhanced angiogenesis, migration, and apoptosis repression by targeting PTEN.

## Discussion

In the present study, it is indicated that miR-214^OE^-Exo promoted the migration, inhibited apoptosis, and improved tubular structure formation under H/SD in HUVECs or NRCMs. Furthermore, we showed that miR-214^OE^-Exo was superior to Ctrl-Exo in protecting cardiac function and inhibiting fibrosis. The protective effects of miR-214^OE^-exo were at least partially mediated through the Akt signaling pathway.

MiRNAs have been proved to be effective therapeutic agent for AMI [5]. The effective and safe delivery system is a key to miRNA-based therapy [28]. Both viral and non-viral delivery systems have been used for target gene suppression. The advantage of viral vectors is high delivery efficiency. However, the toxicity, immunogenicity and tumorigenicity of viral vectors limit clinical application of viral vectors in vivo [29, 30]. Non-viral approaches seem more promising than viral vectors. However, endocytosis during liposome transfection cause endosome rupture or leakage, and trigger adverse inflammation and apoptosis [31]. Recently, studies shows that exosomes are novel RNA delivery tools without endosomal escape [32, 33]. Moreover, exosomes can enter deep tissues when internalized into the multivesicular body (MVB) of the recipient cell, which is then released again and internalized into the MVB of the secondary recipient cell [34, 35]. In conclusion, exosomes have good biocompatibility, high cell uptake efficiency and biological safety, and can be used as an ideal vehicle for miRNAs delivery [15, 36]. For example, MIF-Exo protect heart function by promoting angiogenesis, inhibiting apoptosis, reducing fibrosis by upregulating miR-133a-3p^10^. Sun et al. reported that hypoxic MSC-derived exosome exert cardioprotective effects via the exosomal lncRNAUCA1/miR873-5p/XIAP axis [11].

As lentivirus transduction may also affect the composition of exosomes derived from MSCs, so we choose exosomes isolated from scarmbled-miRNA expressing (negative control lentivirus) MSCs as control group in our study. Therefore, we demonstrated that gene modification of MSCs is a cheap, convenient and efficient method in cardiac repair. The exosomes isolated from miR-214 modified MSCs can be used to animals safely. And we confirmed that miR-214^OE^-Exo was superior to Ctrl-Exo in cardiac repair.

Our data show the anti-apoptotic and pro-angiogenic effects of exosomes released by miR-214^OE^-MSC via phosphatase of Akt signal transduction. The exosome regulates the intracellular signaling pathway, in which exosome miRNAs play important roles in many biological processes. It’s reported that miR-214-3p protected H_2_O_2_ induced cardiomyocytes apoptosis [25]. The deletion of miR-214 gene aggravates cell death induced by ischemia-reperfusion and leads to deterioration of cardiac function. The miR-214 gene knockout led to more severe cell death and decreased left ventricular function, whereas miR-214 is upregulated in ischemic heart and is related to severity of the disease process [37–39]. MiR-214 was reported to be secreted by exosomes of human endothelial cells [26]. This study confirmed that miR-214 modified huMSCs can reduce the area of myocardial fibrosis and improve cardiac function in ischemic hearts. MiR-214 was reported to regulate PTEN in H9C2 cardiomyocytes [38]. To evaluated the effects of miR-214^OE^-Exo, we also assessed the protein expression of PTEN in HUVECs, which can be directed regulated by miR-214. We found that after the treatment of miR-214^OE^-Exo in HUVECs, the expression of PTEN was decreased significantly. These protein-based experiments indicated the potential mechanism of miR-214^OE^-Exo in a more precise manner.

There are some limitations in the current study. Firstly, intramyocardial injection was used to deliver exosomes to target sites. However, this could cause injury of heart and leakage of injected exosomes. Secondly, miR-214^OE^-Exo are non-targeting agents which can also be absorbed by other cells. New targeted delivery methods should be explored to avoid these off-target effects. Thirdly, PTEN may not be the only receptor of miR-214^OE^-Exo to regulate p-AKT signal pathway and further studies are required to validate safety and efficacy of miR-214^OE^-Exo before it becomes a promising cell-free therapy for heart repair in clinical practice.

## Conclusions

Our study verified that miR-214 engineered huMSCs derived exosomes protected heart from ischemia injury through enhancing angiogenesis, promoting cell proliferation, inhibiting apoptosis, and reducing fibrosis in vivo and in vitro. PTEN/AKT signal pathway was one of the potential mechanisms involved in the biological activities of miR-214^OE^-Exo. These findings suggest that the combination of MSC-derived exosomes and miRNAs may be a probable approach for the treatment of cardiovascular disease.

### Electronic supplementary material

Below is the link to the electronic supplementary material.

Supplementary Material 1

## Data Availability

Most of the datasets supporting the conclusions of this article are included within this article and the additional files. The datasets used or analyzed during the current study are available on reasonable request.
