# Supplementary material for: Exosomes derived from mir-214-3p overexpressing mesenchymal stem cells promote myocardial repair
Source: Biomater Res. 2023 Aug 10;27:77. doi: 10.1186/s40824-023-00410-w (PMC10413540; doi:10.1186/s40824-023-00410-w)

**Supplementary table 1. Primer list**

| **Primers** |  | **Sequences (5'–3')** |
| --- | --- | --- |
| hsa-miR-214-3p Forward |  | GACAGCAGGCACAGACA |
| hsa-miR-214-3p Reverse |  | GTGCAGGGTCCGAG |
| PTEN-Forward |  | AATTCCCAGTCAGAGGCGCTATGT |
| PTEN-Reverse |  | GATTGCAAGTTCCGCCACTGAACA |
| GAPDH- Forward |  | GGTGGTCTCCTCTGACTTCAA |
| GAPDH- Reverse |  | GTTGCTGTAGCCAAATTCGTTGT |
| U6 Forward |  | CTCGCTTCGGCAGCACA |
| U6 Reverse |  | AACGCTTCACGAATTTGCGT |
| Cel-miR-39-3p Forward |  | GGGTCACCGGGTGTAAATC |
| Cel-miR-39-3p Reverse |  | GAGAGGAGAGGAAGAGGGAA |

**Supplementary table 2. Sequences list of miR-214a-3p mimics or inhibitor**

|  | **Sequences (5'–3')** |
| --- | --- |
| hsa miR-214-3p mimics | ACAGCAGGCACAGACAGGCAGU |
| hsa miR-214-3p mimics NC | UUUUCCGAACGUUCACGUTT |

**Supplementary Figure 1.** **Comparison of Ctrl-Exos and miR-214^OE^-Exos.**

1. Relative expression of miR-214-3p of different times. (B) The numbers of nanoparticles contained in the isolated Ctrl-Exos and miR-214^OE^-Exos per milliliter. (n = 3 for each group) (C) The protein concentrations of the extracted Ctrl-Exos and miR-214^OE^-Exos per microliter. (n = 3 for each group) All data are mean ± SEM. Statistical analysis was performed with t test. ***P < 0.001.


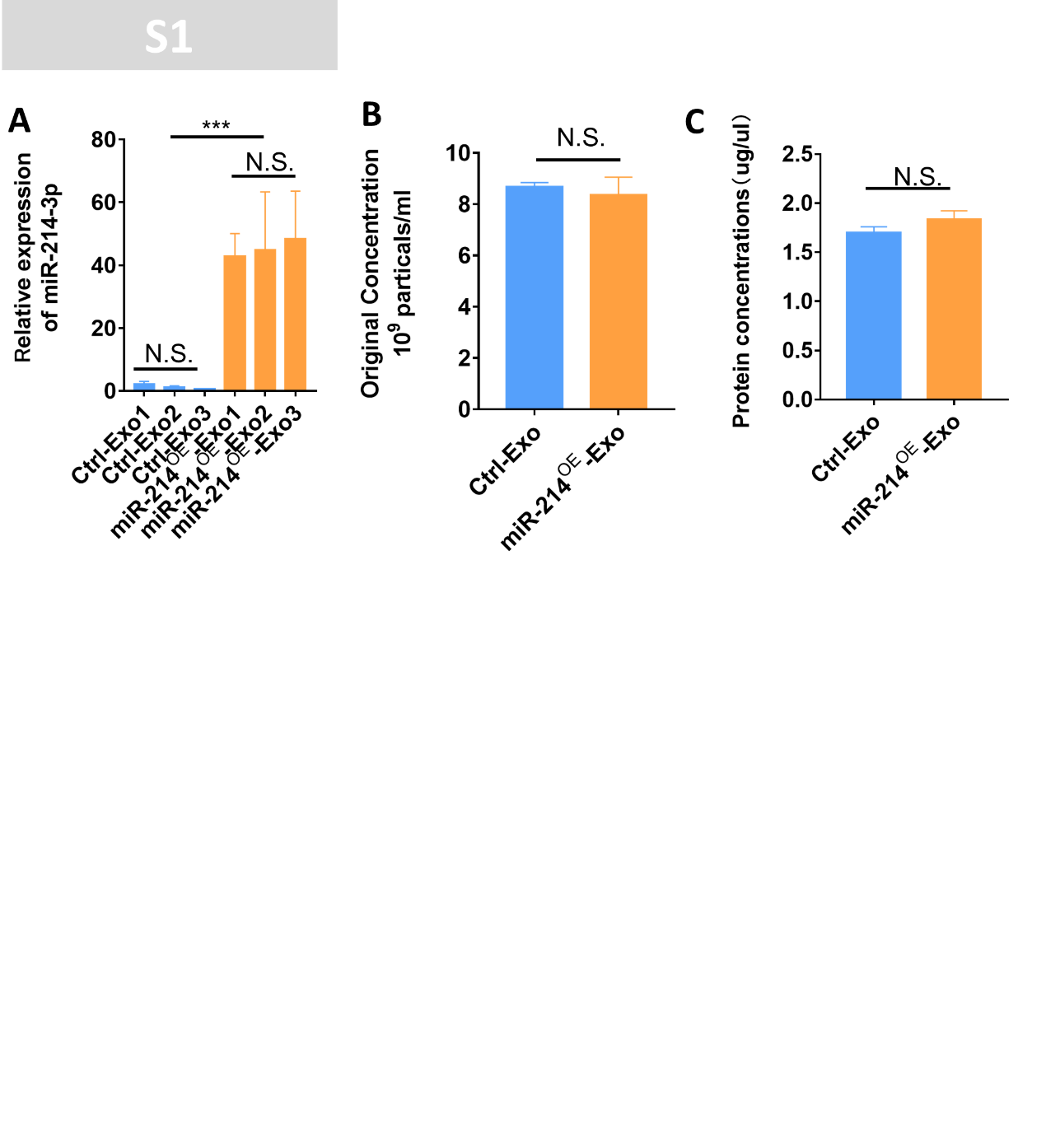


**Supplementary Figure 2. Fluorescent images of intracellular transfer of exosomes labeling Cy3 (A).** **Scale bar =20μm.**


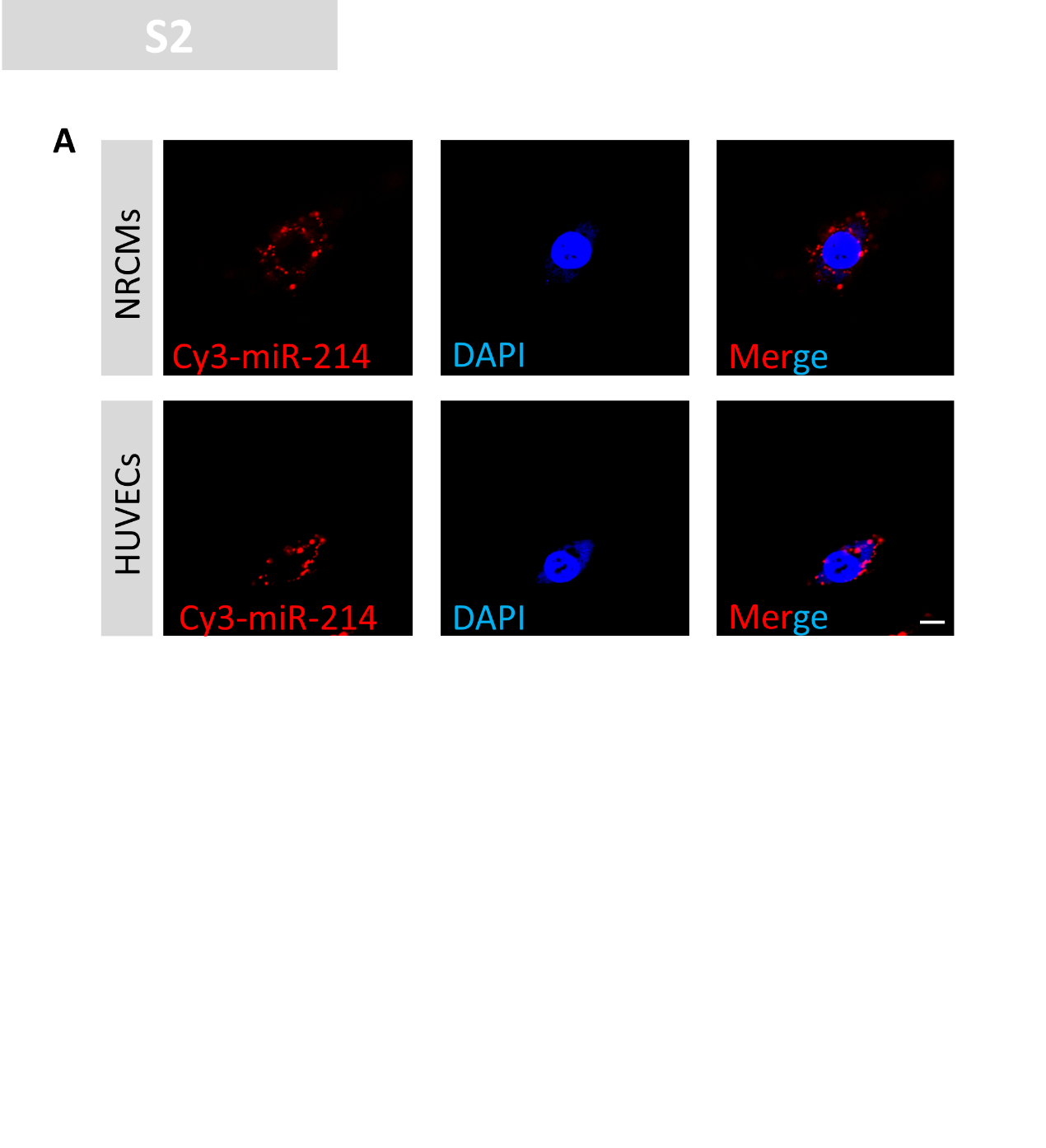


**Supplementary Figure 3. Quantification of incorporated exosomes into cardiomyocytes and endothelial cells.**

(A) The quantification of incorporated exosomes into cardiomyocytes and endothelial cells. Scale bar = 50μm. (n=3 biological replicates for each group). All data are mean ± SEM. Statistical analysis was performed with t test. **P < 0.01.


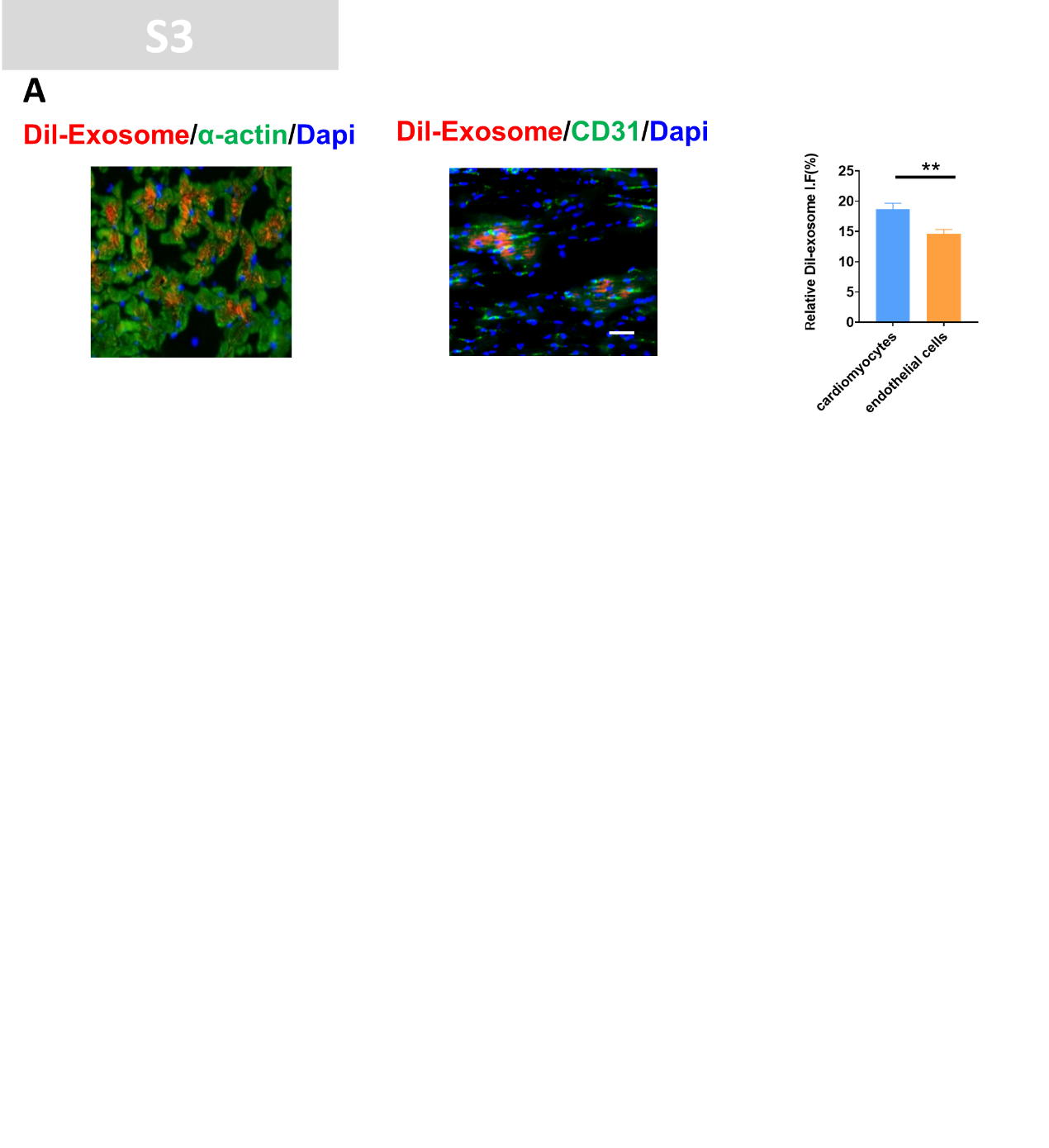


**Supplementary Figure 4. MiR-214^OE^-Exo inhibited the differentiation of cardiac fibroblasts and the occurrence of fibrosis.**

(A) Western blotting image of α-SMA expression level and its semi-quantitative analysis. (n=3 biological replicates for each group). (B) Fluorescent images of collagen expression in different groups and semi-quantitative analysis of their fluorescent images. (n=3 biological replicates for each group). All data are mean ± SEM. Statistical analysis was performed with t test. *P < 0.05, ****P< 0.0001.


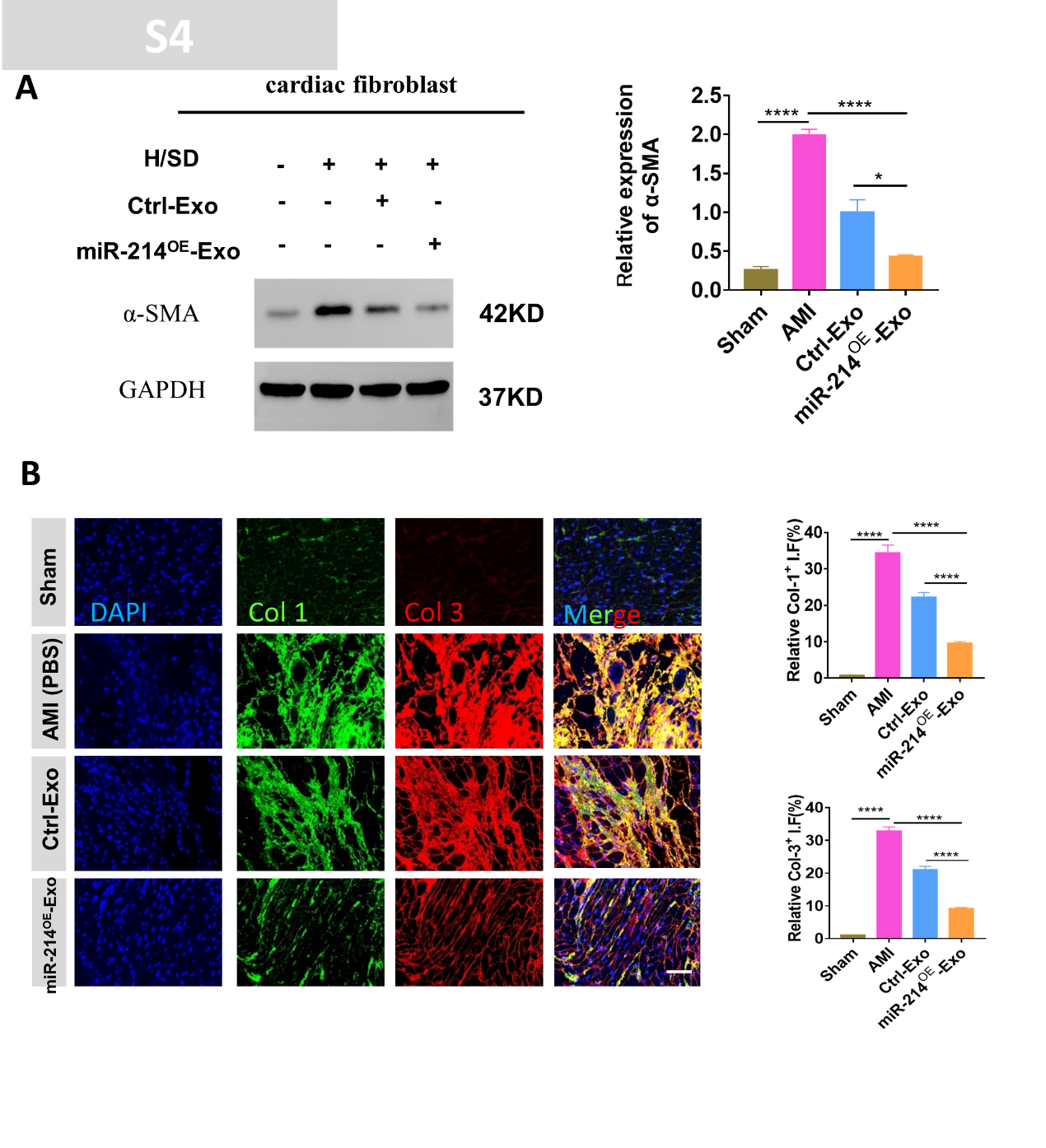


**Supplementary Figure 5. Fluorescent images of cardiac INOS expression levels (A) and the semi-quantitative analysis (B).**


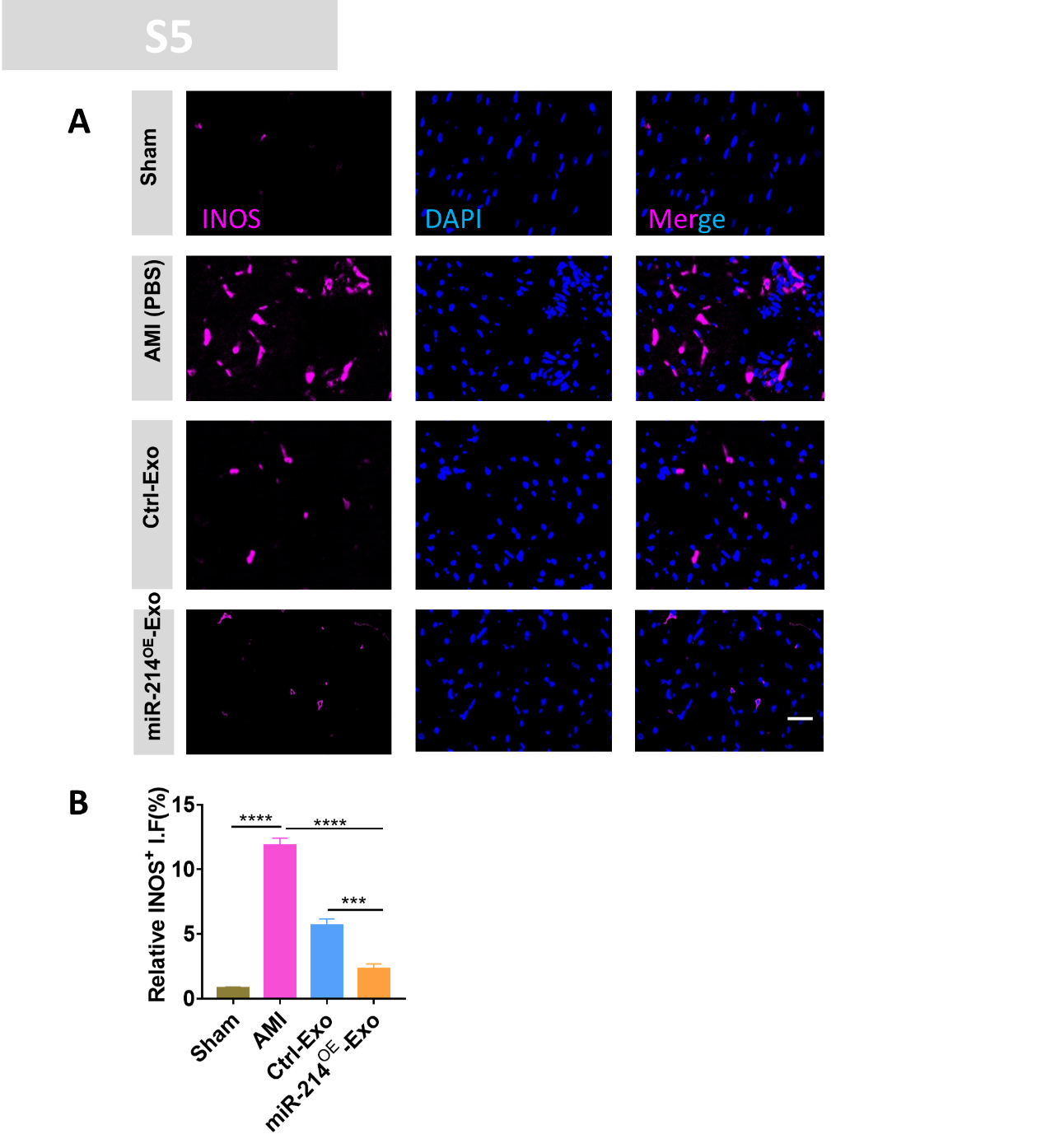


**Supplementary Figure 6. Border zone sections obtained 4 weeks after MI were immunofluorescent stained for Cx43 expression. Red, Cx43; Green, WGA; blue, DAPI-stained nuclei.**

**
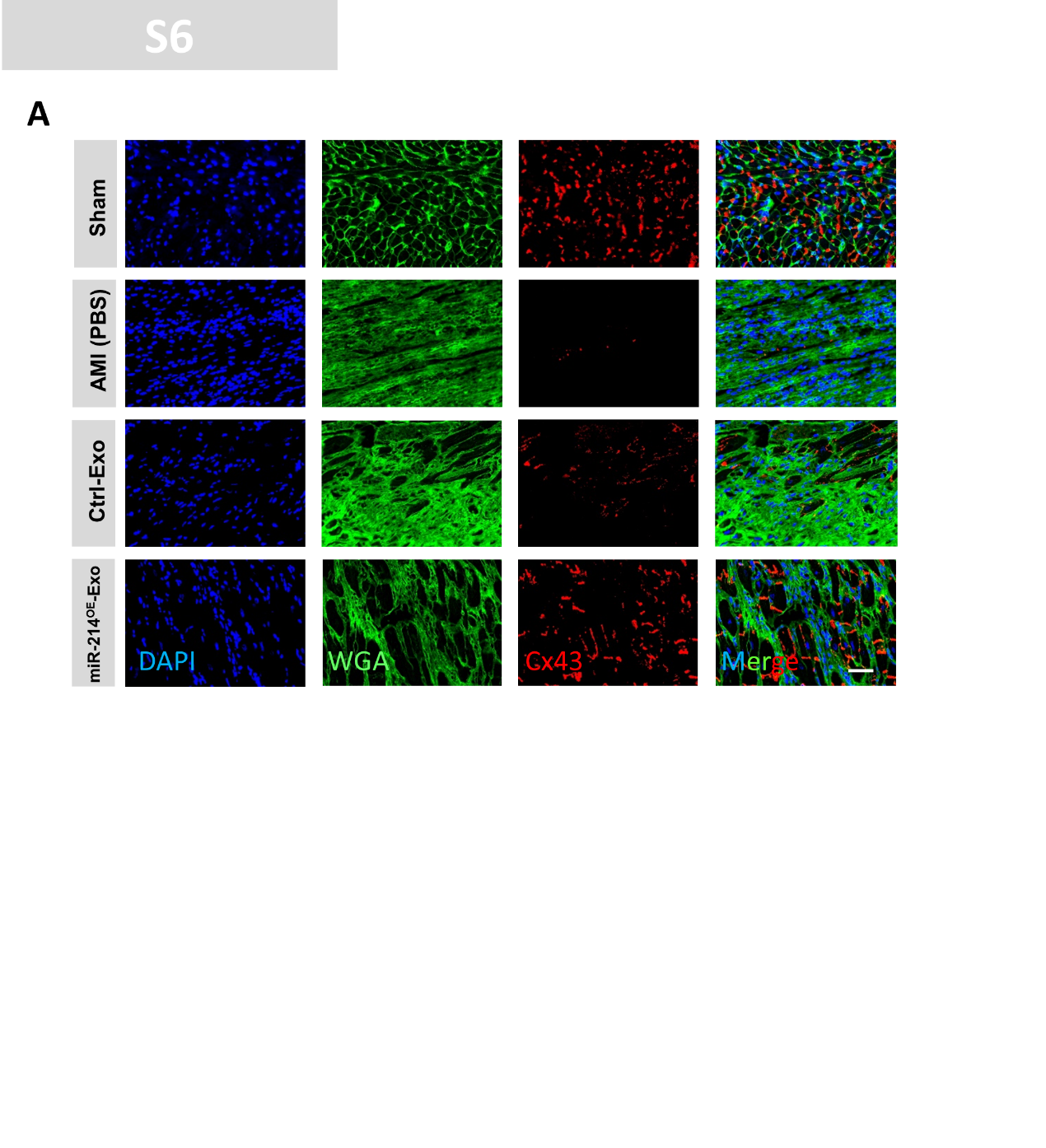
**

**Supplementary Figure 7. Representative immunofluorescence stains of α-actinin in the border area post MI.**


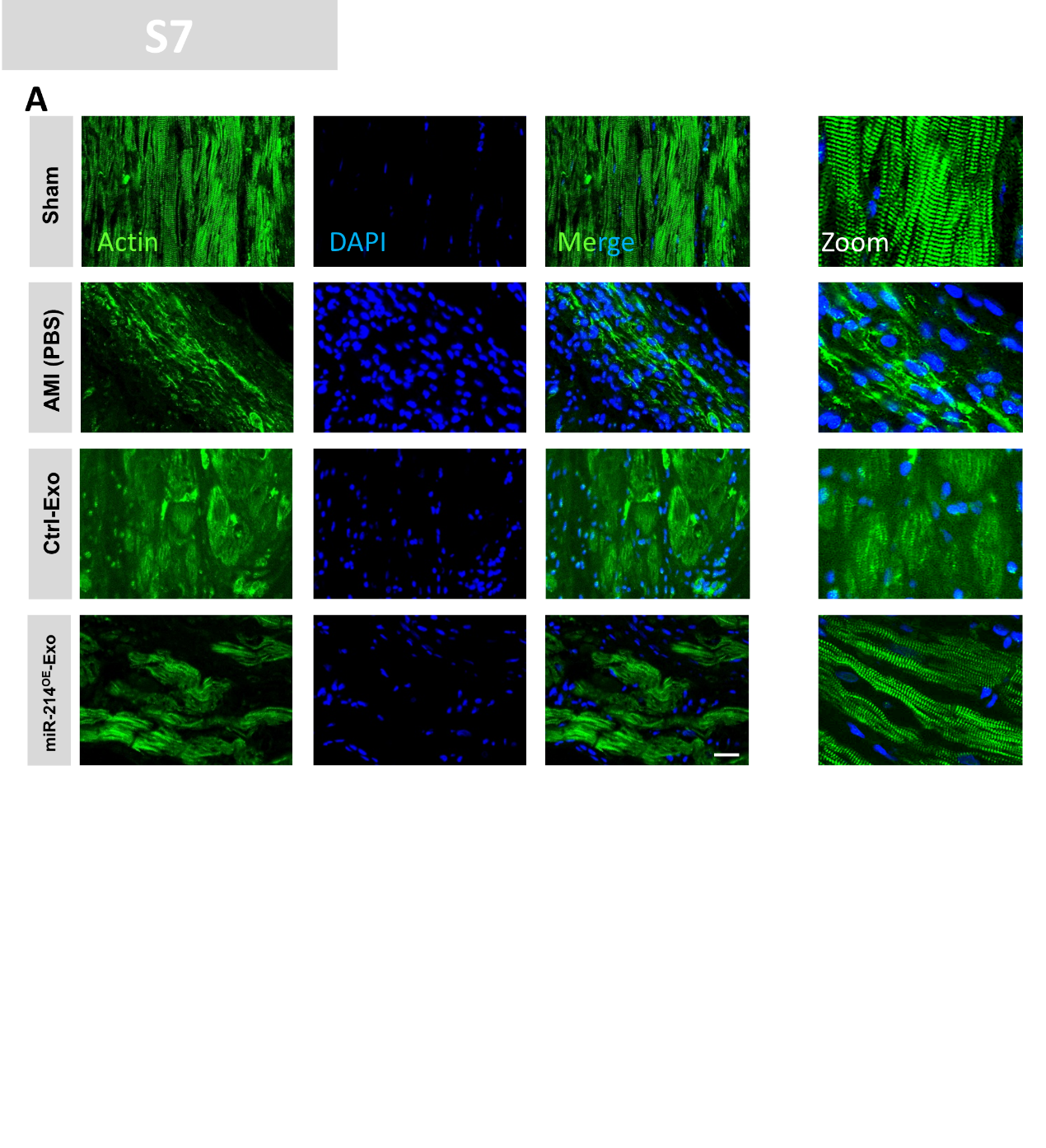


**Supplementary Figure 8. HEK-293T cells and HUVECs were transfected by transfection reagents. (A)** qRT-PCR showed that miR-214 mimics or miR-214 negative controls transfected HEK-293T cells successfully. (n=3 biological replicates for each group). ***P < 0.001. **(B)** Western blot analysis of PTEN in HUVECs after transfection with plasmids containing Vector or PTEN. **(C)** Western blot analysis of PTEN in HUVECs after transfection with Vector or PTEN followed by administration miR-214OE-Exo. (n=3 biological replicates for each group). All data are mean ± SEM. Statistical analysis was performed with t test. ***P < 0.001.


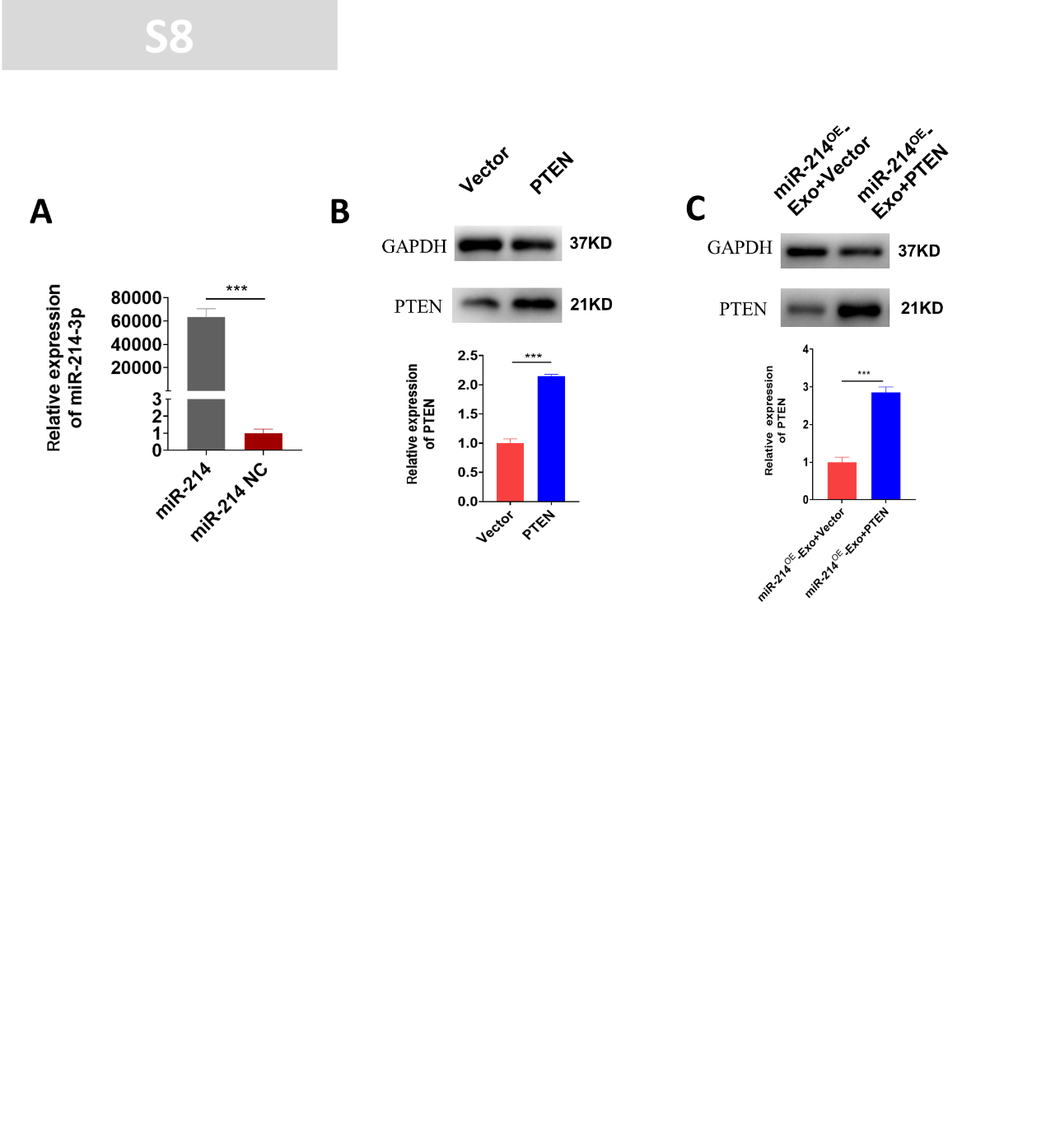

Supplement: Supplementary file 1 — Supplementary Material 1 [file 40824_2023_410_MOESM1_ESM.docx]
